# Negative results of bronchoalveolar lavage fluid metagenomic next-generation sequencing in critically ill patients

**DOI:** 10.3389/fcimb.2022.962283

**Published:** 2022-10-25

**Authors:** Wentao Ma, Yangchao Zhao, Xiaoxiao Lu, Li Zhang, Xiaoxu Ma, Jing Gao, Junna Hou, Qiuhong Liu, Shilong Zhao, Mengying Yao, Lihua Xing

**Affiliations:** ^1^ Department of Respiratory and Critical Care Medicine, The First Affiliated Hospital of Zhengzhou University, Zhengzhou, China; ^2^ Department of Extracorporeal Life Support Center, The First Affiliated Hospital of Zhengzhou University, Zhengzhou, China

**Keywords:** metagenomic next-generation sequencing (mNGS), bronchoalveolar lavage fluid (BALF), negative results, clinical diagnosis, pathological examination

## Abstract

**Objective:**

Reports on negative results of metagenomic next-generation sequencing (mNGS) are scarce. We aimed to explore the diagnostic value of negative results in bronchoalveolar lavage fluid (BALF) mNGS and how to deal with the negative results in patients with severe respiratory disease.

**Methods:**

A retrospective analysis was performed on patients suspected severe community-acquired pneumonia who were admitted to the respiratory intensive care unit of the First Affiliated Hospital of Zhengzhou University from January 2020 to December 2021. According to the final diagnosis as the reference standard, the negative results of mNGS were divided into a true negative group and a false negative group. For enrolled patients, we recorded their demographic data, imaging results, laboratory results, therapeutic processes, and prognoses.

**Results:**

A total of 21 patients were enrolled in this study, including 16 true negative patients and 5 false negative patients. In the true negative group, interstitial lung diseases were the most and neoplastic diseases were following. In addition to mNGS, 9 patients underwent pathological examination, 7 patients were finally diagnosed by medical history, autoantibodies, and point-of-care (POC) ultrasound. 14 patients eventually discontinued antibiotics, 2 patients underwent antibiotic de-escalation, the average interval time of treatment adjustment was 3.56 ± 2.00 days. In the false negative group, the leading missed pathogen was fungi, followed by tuberculosis bacilli. In contrast to 2 patients underwent pathological examination, 3 patients were confirmed by routine microbiological tests.

**Conclusions:**

Negative results of BALF mNGS can help to rule out infection, but missed diagnoses may also exist. It should be re-evaluated with other clinical informations. Pathological examination or repeated mNGS may be viable options when the diagnosis cannot be confirmed.

## Introduction

Respiratory failure is a common cause of admission to the ICU, and pulmonary infection is the most common reason for respiratory failure. Rapid identification of pathogens is the key to successful treatment of respiratory failure. Compared with conventional microbial detection, metagenomic next-generation sequencing (mNGS) identifies all the nucleic acids of microorganisms contained in the sample, exhibiting the advantages of time consuming and high detection rate (Gu et al., 2019). In addition, studies have found that the application of antibiotics has less impact on mNGS than traditional culture (Miao et al., 2018). The feasibility and effectiveness of bronchoalveolar lavage fluid (BALF) mNGS have been demonstrated, and one multicentre prospective study showed that 40% of patients underwent treatment adjustment based on their mNGS test results (Zhou et al., 2021). Negative results of BALF mNGS are the situations that we will encounter in clinical practice. This study retrospectively investigated the clinical information of patients with negative results of BALF mNGS, aimed to explore the clinical significance of negative results and how to further handle the situation when facing negative results.

## Materials and methods

### Study design

A retrospective analysis was performed on patients suspected of severe community-acquired pneumonia who were admitted to the respiratory intensive care unit (RICU) of the First Affiliated Hospital of Zhengzhou University from January 2020 to December 2021. Inclusion criteria: 1. At least 18 years old. 2. The initial diagnosis on admission was suspected of severe community-acquired pneumonia (CAP), which meets the diagnostic criteria of severe CAP (Respiratory Society of Chinese Medical Association, 2016). 3. Antibiotics were used empirically after admission. 4. Fiberoptic bronchoscopy was performed within 48 hours after admission, and BALF was collected for mNGS with negative results.

Exclusion criteria: 1. Infection of sites other than the lung occurred during ICU hospitalization. 2. BALF was not simultaneously underwent routine microbiological tests. 3. BALF specimens failed to pass mNGS quality control or unqualified BALF samples, eligibility criteria of BALF: under the low-power microscope, squamous epithelial cells constitute <1% of all cells (excluding red blood cells), the proportion of columnar epithelial cells <5% and red blood cells<10% (except trauma and bleeding). 4. clinical data was incomplete.

For each patient who met the criteria, we recorded their demographic data, comorbidities, imaging results, laboratory results, therapeutic processes, and prognoses. The patients were divided into a true negative group and a false negative group according to the final diagnosis as the reference standard.

### The definition of negative results of BALF mNGS

Negative results of BALF mNGS are defined as follows: results of BALF mNGS don’t provide significant etiological informations for antibiotic application or only provided some pathogens identified as background microorganisms.

### mNGS of BALF

The lesion sites which were the most rapid progression or the most severe determined by chest imaging were selected for lavage. Emerging or gradually progressive lesions are selected in the localized lesions. The middle lobe of the right lung or lingual segment of left lung are selected in the diffuse lesions. Bronchoalveolar lavage was performed 3-5 times and the volume of bronchoalveolar lavage was 60-120ml. The collected BALF samples were divided into two parts and sent to laboratory within 2 hours for mNGS and conventional microbial detection. The mNGS process included specimen preprocessing, nucleic acid extraction, library construction, sequencing, and information analysis. RNA extraction and sequencing procedures were applied if an RNA viral infection was suspected. All sequences had been uploaded into EMBL ena database with accession ID is PRJEB55113.

### Statistical analysis

SPSS 26.0 software was applied for statistical analysis. The quantitative data were expressed as the mean ± standard deviation or the median and quartile spacing for data with normal distribution and non-normal distribution, respectively. Independent sample T tests were used to compare the inter group differences for normally distributed data, while Mann–Whitney U tests were used for non-normally distributed data. The counting data were expressed as frequencies and percentages, and the Fisher’s exact probability method was used to compare independent samples. *P*<0.05 was considered statistically significant, and all tests were two-tailed tests.

## Results

### Patient characteristics

During the study period, a total of 21 patients were eventually included in the final analysis, with 16 in the true-negative group and 5 in the false-negative group. In the former group, the mean age was 58.69 ± 14.09 years; 43.75% patients (7/16) were male; and 68.75% patients (11/16) had comorbidities, including diabetes, hypertension, chronic obstructive pulmonary disease, oesophageal cancer, etc. In the latter group, the mean age was 53.20 ± 14.08 years; 40.00% patients (2/5) were male; and 80.00% patients (4/5) had comorbidities, containing diabetes, rheumatoid arthritis, chronic obstructive pulmonary disease, and leukaemia. There were no significant differences in inflammation biomarkers, chest imaging, and oxygenation index between the true-negative group and the false-negative group. Further details are shown in **Table 1**.

**Table 1:** Clinical characteristics of enrolled patients.

	True-negative group	False-negative group		*P* value
Number of patients	16	5		
Age (years)^a^	58.69±14.09	53.20±14.08		0.456
Gender^*^				1.000
Male	7 (43.75%)	2 (40.00%)		
Female	9 (56.25%)	3 (60.00%)		
Comorbidities^*^				1.000
Yes	11 (68.75%)	4 (80.00%)		
No	5 (31.25%)	1 (20.00%)		
Inflammation biomarker^a^				
WBC (^10^9^/L)	10.61±4.09	9.38±4.27		0.569
CRP (mg/L)	67.97±50.64	52.52±38.96		0.541
PCT (ug/L)	0.54±0.39	0.46±0.49		0.696
Abnormality on chest radiograph^*^				1.000
Unilateral lesion	3 (18.75%)	1 (20.00%)		
Bilateral lesion	13 (81.25%)	4 (80.00%)		
Oxygenation index (mmHg)^a^	163.75±41.30	195.40±20.43		0.119
mNGS^*^				1.000
DNA	14 (87.50%)	5 (100.00%)		
DNA+RNA	2 (12.50%)	0		
Conventional microbal tests^*^				0.008
Negative	16 (100.00%)	2 (40.00%)		
Positive	0	3 (60.00%)		
Length of stay (days)^a^				
In ICU (mean±SD)	10.56±3.27	9.60±4.78		0.611
In hospital (mean±SD)	17.81±4.83	18.00±10.37		0.955
Hospital death^*^	3 (18.75%)	1 (20.00%)		1.000

a The values are given as the mean and the standard deviation. ^*^The values are given as the number of cases, with the percentage in parentheses.

### The true negative group

**Figure 1:**
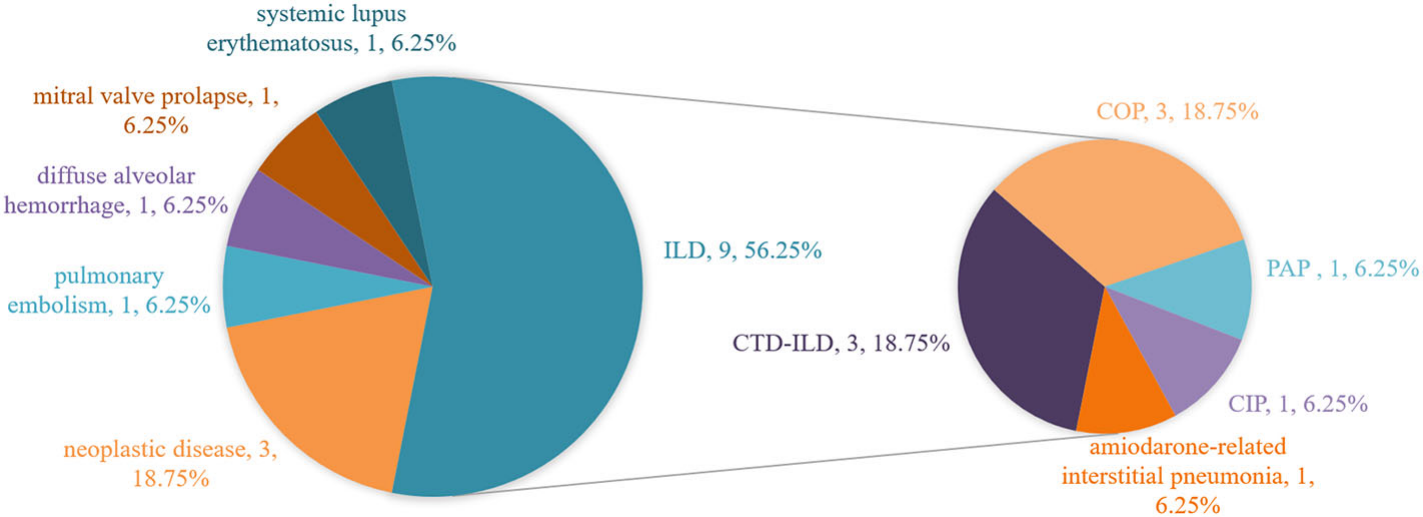
Final diagnoses in the true negative group. ILD, interstitial lung disease; CTD-ILD, connective tissue disease-related interstitial lung disease; COP, cryptogenic organizing pneumonia; PAP, pulmonary alveolar proteinosis; CIP, checkpoint inhibitor pneumonitis.

Final diagnoses of the true negative group are shown in **Figure 1**, there were 9 cases of interstitial lung disease, including 3 connective tissue disease-related interstitial lung disease (CTD-ILD, P9, P10, P14), 3 cryptogenic organizing pneumonia (COP, P4, P6, P8), 1 pulmonary alveolar proteinosis (PAP, P3), 1 amiodarone-associated interstitial pneumonia(P15), and 1 checkpoint inhibitor pneumonitis (CIP, P5). 3 patients had concurrent neoplastic disease, including 2 with lung adenocarcinoma (P2, P11) and 1 with mucosa-associated lymphoid tissue lymphoma(P13). Others included 1 pulmonary embolism(P1), 1 diffuse alveolar hemorrhage(P7), 1 pulmonary edema caused by mitral valve prolapse(P16), and 1 systemic lupus erythematosus(P12).

**Figure 2:**
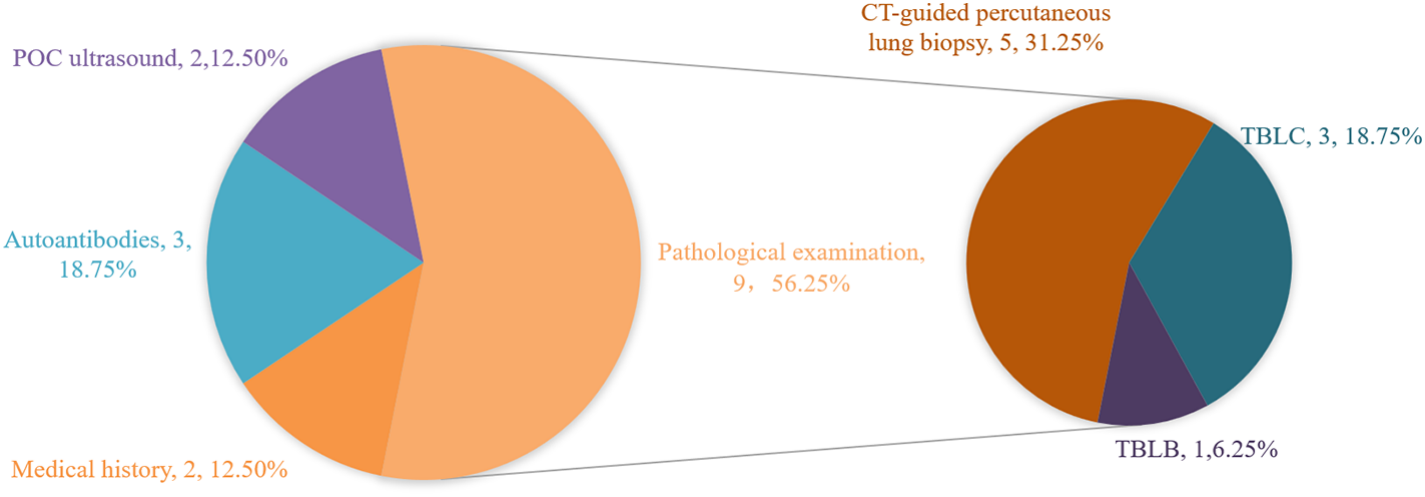
Main diagnostic bases except BALF mNGS in the true negative group. POC ultrasound, point-of-care ultrasound; TBLB, transbronchil lung biopsy; TBLC, transbronchial lung cryobiopsy.

**Figure 3:**
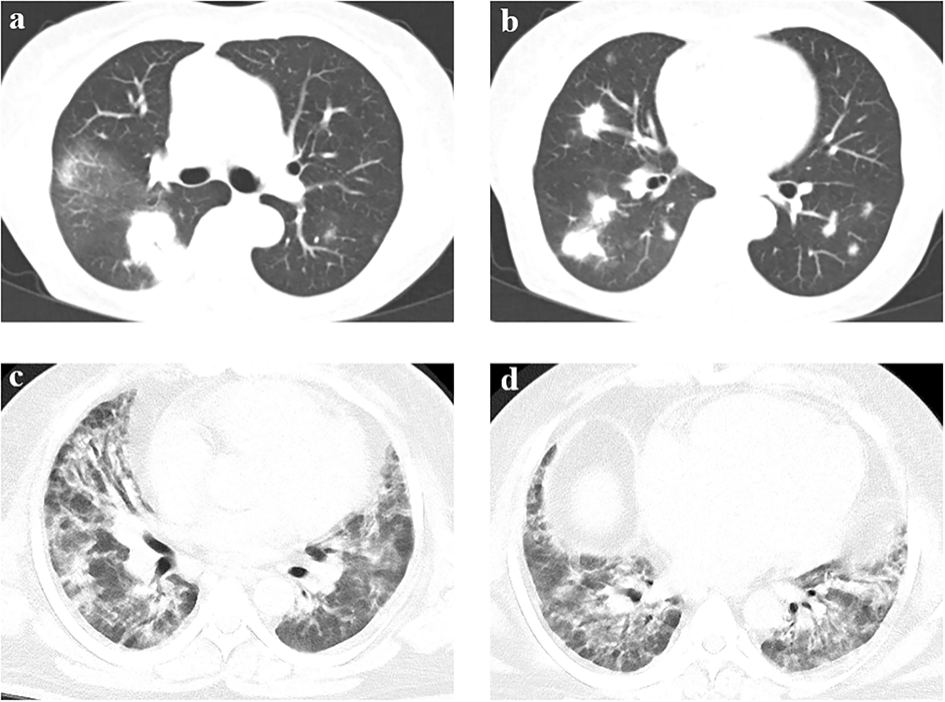
Chest CT of two representational cases. **(A, B)** A 71-year-old female patient was admitted to the RICU with a chief complaint of fever with cough, sputum and chest tightness for ten days. Chest CT showed bilateral multiple-tubercle shadow and consolidations after admission. The result of BALF mNGS was negative in the initial assessment. Then CT-guided percutaneous lung biopsy was performed. Pathology of percutaneous lung biopsy revealed chronic inflammation of lung tissue with interstitial fibrosis, showing organized pneumonia changes. The final diagnosis was cryptogenic organizing pneumonia. **(C, D)** A 43-year-old male patient was admitted to the RICU with a chief complaint of shortness of breath for three weeks and fever for 2 days. Chest CT showed diffuse patchy shadow with local interstitial changes over bilateral lung fields. The result of BALF mNGS was negative in the initial assessment. The results of myositis autoantibodies showed that anti-PM-Scl75 antibody(+++), anti-PM-Scl100 antibody(++) and anti-Ku antibody(+++). The final diagnosis was idiopathic inflammatory myopathy related interstitial lung disease.

In addition to mNGS, main diagnostic bases of the true negative group are shown in **Figure 2**. A total of 9 patients underwent pathological examination, with 5 patients underwent CT-guided percutaneous lung biopsy (P8, P11, P12, P13, P14) and 3 patients underwent transbronchial lung cryobiopsy (TBLC, P2, P4, P6). Among these 3 patients, 1 patient underwent TBLC during the second tracheoscopy(P4). Autoantibody tests provided diagnostic clues in 3 patients (P7, P9, P10), except common autoantibodies, P9 and P10 made the diagnosis by screening for myositis autoantibodies. Furthermore, the diagnosis was clarified by medical history for 2 patients (P5, P15) and point-of-care (POC) ultrasound for the other 2 patients (P1, P16). Chest CT images of two representational cases are shown in **Figure 3**.

**Figure 4:**
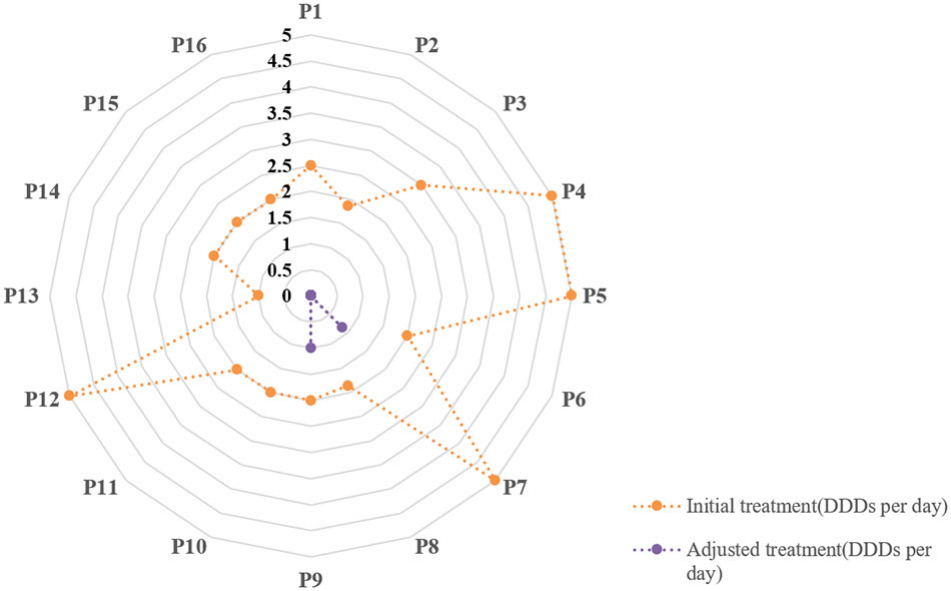
The adjustment of treatments in the true negative group. The defined daily doses (DDDs) per day were introduced to reflect daily antibiotic use for each patient.

**Figure 5:**
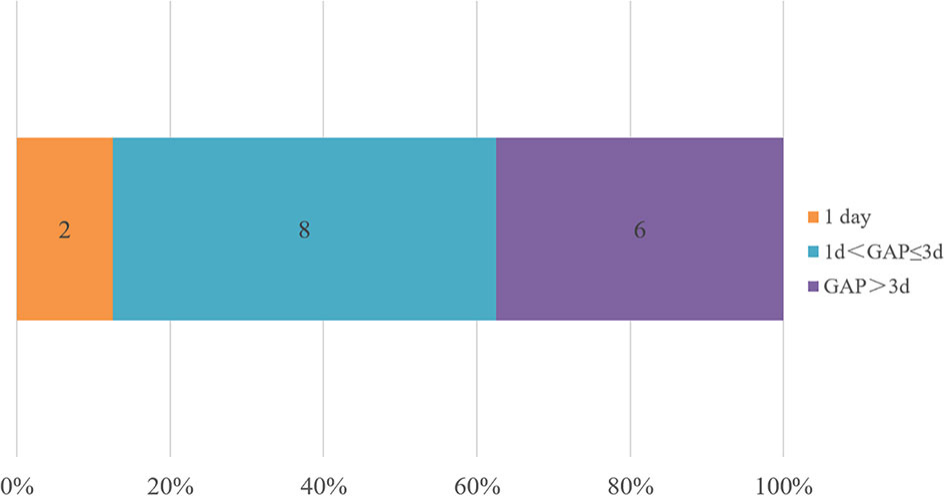
The adjustment time in the true negative group. We defined GAP as the interval time between when the mNGS results were received and the time when the antibiotic treatment regimen was adjusted. Less than 1 day is counted as 1 day.

The adjustments of treatments in the true negative group are shown in **Figure 4**. The defined daily doses (DDDs) per day were introduced to reflect the daily antibiotic use for each patient. In the true negative case group, antibiotics were eventually discontinued in 87.5% patients (14/16) and were downgraded in 12.5% patients (2/16). We defined GAP as the interval time between when the mNGS results were confirmed and the time when the antibiotic treatment regimen was adjusted. The GAP of the true negative group are shown in **Figure 5**. It indicated that the mean GAP was 3.56 ± 2.00 days, GAP of 1 day in 12.5% patients (2/16), GAP of greater than 3 days in 37.5% patients (6/16) and the remaining patients had a GAP of between 1 day and 3 days.

### The false negative group

Final diagnoses and diagnostic bases of the false negative group are shown in **Table 2**. In the false negative group, the missed pathogens included RNA viruses in 1 patient and Rhizopus microsporus, Aspergillus, Cryptococcus, Mycobacterium tuberculosis in another patient. 2 patients underwent a pathological examination by percutaneous lung biopsy, with 1 patient had mNGS from lung tissue. 3 patients were confirmed by routine microbiological tests.

**Table 2:** Final diagnoses and diagnostic bases in the false negative group.

Patient no.	Final diagnosis	Main evidences (except BALF mNGS)	Notes
17	HRSV-Caused Pneumonia	pathogenic antibody	
18	Pulmonary rhizomycosis	mNGS of lung tissues+pathological examination	percutaneous lung biopsy
19	Pulmonary aspergillosis	fungal cultures of BALF	
20	Pulmonary cryptococcosis	pathological examination	percutaneous lung biopsy
21	pulmonary tuberculosis	acid-fast staining of BALF	

HRSV, Human Respiratory Syncytial Virus.

## Discussion

The mortality of lung infection is the highest among the infectious disease in the world, which is caused by treatment failure due to drug resistance to pathogenic microorganisms and the limitations of traditional etiological tests in identifying infectious pathogens. Delays in diagnosis may hinder precision treatment and further lead to more resistant pathogens, increase medical costs and poor prognosis. However, mNGS does not rely on traditional microbial isolation and culture, using high-throughput sequencing as a tool, with the characteristics of high efficiency, unbiased and extensive coverage, providing more comprehensive and objective information. Most mNGS platforms can obtain pathogenic results within 24h, which greatly shortens the detection time of pathogens, and can help clinicians to evaluate the condition and guide clinical decisions more timely. Therefore, it has been more and more favored by clinicians in recent years. Negative results are the situation that we may encounter in the clinical practice. This study retrospectively investigated the clinical information of patients with negative results of BALF mNGS, aimed to explore the clinical significance of negative results and how to further handle the situation when facing negative results.

In this study, patients admitted with an initial diagnosis of severe CAP and timely submitted for BALF mNGS were included and grouped according to the final diagnosis results, including 16 patients in the true-negative group and 5 patients in the false-negative group. Based on these negative results, 14 patients eventually discontinued antibiotics, 2 patients underwent antibiotic de-escalation, while 5 patients had a missed diagnosis. In the true-negative group, the number of cases finally diagnosed with interstitial lung disease ranked first, accounting for 56.25%, of which the number of CTD-ILD and COP cases accounted for 18.75% respectively. The number of neoplastic diseases ranked second, accounting for 18.75%, and the pathological type was dominated by lung adenocarcinoma. In the false-negative group, the leading missed pathogen was fungi, accounting for 60%, including 1 case of rhizopus (P18), 1 case of aspergillus (P19), 1 case of cryptococcus (P20), followed by tuberculosis bacilli (P21).

False negative results may occur when the pathogenic load in the sample is too low to be detected or the sequence number is below the reported threshold. Chen et al. summarized the factors that may account for false negative results of mNGS include the following: (a) inadequate sequencing depth; (b) prior antibiotic usage; (c) high host genome background and low microbial biomass of the true pathogens; (d) strict filtering strategy for mNGS results (Chen et al., 2020). P17 did not have RNA detection procedures performed. Missed diagnoses in P19, P20 and P21 were due to the difficulty in breaking the cell wall of fungus and tuberculosis bacilli. Rhizopus was not detected from the BALF mNGS of P18, the reason maybe that Rhizopus is difficult to remove from lesions by lavage and usually requires tissue samples to diagnose (Tsyrkunou et al., 2014).

This study shows that negative results can help to rule out infection, but missed diagnoses may also exist. This conclusion is in agreement with previous studies. One study involving 32 critically ill patients showed that 2 BALF mNGS-negative patients were eventually diagnosed with dermatomyositis and organizing pneumonia (Li et al., 2019). Another multicentre prospective study found that antibiotic degradation was performed in 19 patients based on negative BALF mNGS results, while tuberculosis was missed in 5 subjects (Zhou et al., 2021).

What can we do when confront with the negative results of BALF mNGS? As we know, mNGS can provide results within 24 hours, which is faster than conventional microbial tests and pathological examination, and even some blood testings. In addition, **Table 1** shows that there are no significant differences in inflammation biomarkers, chest imaging, and oxygenation index between the true-negative group and the false-negative group. Therefore, the clinical information for analysis is limited at the time of obtaining negative results of BALF mNGS. This may explain the situation of **Figure 5** that only 12.5% of patients were clearly diagnosed within 1 day and 62.5% of patients were clearly diagnosed within 3 days in the true negative group. In our study, the diagnostic evidence was divided into pathological and nonpathological, except for mNGS. These nonpathological evidence included patient’s medical history, autoantibody, point-of-care ultrasound and routine microbiological tests.

In this study, P5 and P15 were diagnosed with CIP and amiodarone-related interstitial pneumonia, respectively, by reviewing their previous treatment process, indicating that medical history often contains a wealth of information, which may be the breakthrough point of disease diagnosis. At present, the medical history of some patients is too simple to reflect the continuous process of the disease. The clinical features of autoimmune diseases are complex and variable, and the same clinical manifestations and imaging changes as those of pulmonary infectious diseases may occur when the lung is affected. Autoantibody testing is an important tool for diagnosis and differential diagnosis (Rose, 2008). P9 and P10 were tested for autoantibodies after their negative results of BALF mNGS. However, P7 was screened for autoantibodies earlier, so the adjustment time of treatment was earlier than P9 and P10. POC ultrasound is considered as the fifth physical examination to assess patient’s condition in real time (Narula et al., 2018). In our study, the possibility of pulmonary embolism was considered in P1 through POC ultrasound and was confirmed by CT pulmonary arteriography, and P16 was observed to have posterior mitral valve prolapse into the left atrium. Although the patients’ treatments were quickly adjusted, it was possible that mNGS would not have been necessary if these patients were evaluated with POC ultrasound in advance.

As previously described, routine microbiological tests can help to reduce the missed diagnosis of mNGS. Wang et al. found that 2 cases missed by mNGS included one with pulmonary cryptococcosis and one with pulmonary aspergillosis among 21 cases of fungal pneumonia (Wang et al., 2020). In the former, the capsular polysaccharide antigen detection was positive. In the latter, both the culture and galactomannan (GM) test results were positive. The reasons are considered to be related to the low efficiency of nucleic acid extraction due to the difficulty and insolubility of the fungal cell wall, clinicians should consider the results of traditional methods when selecting mNGS. In the infection of mycobacterium tuberculosis, mNGS is difficult to detect because the intracellular growth characteristics of tuberculosis, less nucleic acid is released outside the cell. Zhou et al. showed that in the identification of active cases, the sensitivity of mNGS was 44%, higher than that of traditional detection methods (29%), and the diagnostic rate can reach 60% under the combination of mNGS and Xpert (Zhou et al., 2019). mNGS is a complementary, but not a substitute test for conventional microbial methods. Peng et al. deemed that when conventional microbial tests were comprehensive enough, there was no statistically significant difference in the diagnostic ability of mNGS, as compared to the conventional microbial tests, the combination of mNGS and conventional microbial tests may be a better diagnostic strategy (Peng et al., 2021).

Pathological examination is considered as the gold standard for diagnosis. Previous studies have shown that lung biopsy can be considered if the initial noninvasive examination does not provide clear clues and the risk of empirical treatment is too high or empirical treatment fails (Palakshappa & Meyer, 2015). The methods of lung biopsy include surgical lung biopsy, percutaneous lung biopsy, bronchoscopy, etc. TBLC is a new method with the advantages of less trauma, large specimens, high quality and fewer complications (Babiak et al., 2009). A variety of lung biopsy methods were observed in this study, 7 patients adopted CT-guided percutaneous lung biopsy, 3 patients underwent TBLC and 1 patient employed TBLB. The opportunity of lung biopsy was also different. Considering that pathological examination often takes a long time, it may be helpful to perform BALF mNGS and pathological examination at the same time during the operation of endoscopy examination for early diagnosis.

Opinions are contradictory on whether mNGS should be repeated if the initial result is negative. An expert consensus mentioned that if the possibility of infection cannot be ruled out, resampling and repeated testing were recommended if necessary (Editorial Board of Chinese Journal of Infectious Diseases, 2020). However, Filkins et al. believed that repeated mNGS was not advised for negative initial test results because repeated testing did not increase the positive possibility (Filkins et al., 2020). In this study, Rhizopus was not detected from the BALF mNGS of P18, whereas both mNGS and the histopathological examination were positive in the lung tissue, suggesting that lung tissue may be helpful in the diagnosis of the diseases when patients requiring mNGS retesting. A study of 2018 found that mNGS can be used for pathogen detection in lung tissue and may have potential advantages in speed and sensitivity compared with traditional culture (Li et al., 2018). On the other hand, Yang et al. discovered that no difference was observed in the sensitivity and specificity of the diagnosis of pulmonary fungal infection between lung biopsy and BALF (Yang et al., 2021).

In the face of negative results of BALF mNGS, first of all, we should combine medical history, autoantibody, point-of-care ultrasound for differential diagnosis. The interval time may be shortened from the mNGS results were obtained to the antibiotic treatments were adjusted if the clinical informations mentioned above could be carefully reviewed prior to mNGS. Attention should be focused on the results of routine microbiological tests if pulmonary infection cannot be excluded based on mNGS. Pathological examination or repeated mNGS may be helpful when the diagnosis remains elusive.

We acknowledge that this study has some limitations. First, this was a single-centre retrospective observational study with a small sample size. Second, the study was not a randomized controlled study. Third, the results of BALF mNGS came from different laboratories, and sequencing differences may affect the results.

## Conclusion

Negative results of BALF mNGS can help to rule out infection, but missed diagnoses may also exist. It is a wise decision to combine mNGS with other clinical informations including medical history, autoantibody, point-of-care ultrasound, routine microbiological tests. Pathological examination or repeated mNGS may be viable options when the diagnosis cannot be confirmed.

## Data availability statement

The datasets presented in this study can be found in online repositories. The names of the repository/repositories and accession number(s) can be found below: All sequences had been uploaded into EMBL ena database with accession ID PRJEB55113.

## Ethics statement

The studies involving human participants were reviewed and approved by Ethics Committee of the First Affiliated Hospital of Zhengzhou University. Written informed consent for participation was not required for this study in accordance with the national legislation and the institutional requirements.

## Author contributions

WM, YZ, MY, and LX conceived the study. YZ, XM, JG, QL, JH and SZ collected the cases and related clinical information. XL and LZ collected and analyzed the data. WM wrote the draft. All authors contributed to the article and approved the submitted version.

## Conflict of interest

The authors declare that the research was conducted in the absence of any commercial or financial relationships that could be construed as a potential conflict of interest.

## Publisher’s note

All claims expressed in this article are solely those of the authors and do not necessarily represent those of their affiliated organizations, or those of the publisher, the editors and the reviewers. Any product that may be evaluated in this article, or claim that may be made by its manufacturer, is not guaranteed or endorsed by the publisher.
